# Predicting Momentary Suicidal Ideation From Smartphone Screenshots Using Vision-Language Models: Prospective Machine Learning Study

**DOI:** 10.2196/90581

**Published:** 2026-04-08

**Authors:** Ross Jacobucci, Wenpei Shao, Veronika Kobrinsky, Brooke Ammerman

**Affiliations:** 1 Center for Healthy Minds, University of Wisconsin–Madison, 625 W Washington Ave, Madison, WI, 53703, United States, 1 (608) 263-6321; 2 Department of Psychology, University of Wisconsin–Madison, Madison, WI, United States

**Keywords:** digital phenotyping, suicide, passive sensing, phone use, smartphone, foundation models

## Abstract

**Background:**

Passive smartphone sensing shows promise for suicide prevention, but behavioral metadata (GPS, screen time, and accelerometry) often lacks the contextual information needed to detect acute psychological distress. Analyzing what people actually see, read, and type on their phones—rather than just usage patterns—may provide more proximal signals of risk.

**Objective:**

This study aimed to test whether vision-language models (VLMs) applied to passively captured smartphone screenshots can predict momentary suicidal ideation (SI).

**Methods:**

Seventy-nine adults with past month suicidal thoughts or behaviors completed ecological momentary assessments (EMA) over 28 days while screenshots were captured every 5 seconds during active phone use. We fine-tuned open-source VLMs (Qwen2.5-VL [Alibaba Cloud], LFM2-VL [Liquid AI]), and text-only models (Qwen3 [Alibaba Cloud]) to predict SI from screenshots captured in the 2 hours preceding each EMA. We evaluated performance with temporal and subject holdouts.

**Results:**

The analytic sample comprised 2.5 million screenshots from 70 participants. Temporal holdout models achieved strong discrimination at the EMA level (AUC=0.83; AUPRC=0.77), with image-based models outperforming text-only models (AUC=0.83 vs 0.79; 95% CI 0.003-0.07). Subject holdout generalization was near chance (AUC≈0.50), though a simple lexical screening method retained modest discrimination (AUC=0.62). Smaller models performed comparably to larger models, supporting feasible on-device deployment.

**Conclusions:**

Screen content predicts short-term SI with clinically meaningful accuracy when models are personalized but does not generalize across individuals. These findings support a 2-stage clinical architecture, coarse lexical screening for new patients, with personalized VLM-based monitoring after a calibration period. On-device inference may enable privacy-preserving deployment.

## Introduction

### Background

Digital mental health increasingly aims to detect short-term changes in risk and deliver support precisely when needed via just-in-time adaptive interventions (JITAIs) [1]. Continuous, unobtrusive data streams from smartphones and wearables can provide objective, high-frequency indicators of psychological states, including mobility, communication, sleep, heart rate variability, and device usage. These passive sensing modalities are well-suited to the hour-to-hour fluctuations characteristic of suicidal ideation (SI) [2,3]. In suicide-focused passive sensing, feasibility has been demonstrated in acute settings using phone-derived measures [4], with additional evidence that combining ecological momentary assessment (EMA) with passive data improves short-horizon prediction of SI [3] and that intensive longitudinal modeling can track SI trajectories over time in demanding real-world contexts [5,6].

Over the past few years, the literature has expanded across sensing modalities, analytic approaches, and clinical settings. Work includes GPS-based detection of risk among high-risk adolescents [7], noninvasive speech analysis in emergency care [8], physiological signals from wearables for imminent risk identification [9-12], and smartphone-based monitoring over weekly horizons [13]. Methodological advances span transformer-based emotion forecasting [14] and multimodal, contrastive pipelines that fuse active and passive signals [15]. Recent studies from our group further suggest that on-screen text and content collected passively may relate directly to suicide risk, complementing traditional sensors [16-18]. Taken together, systematic reviews conclude that suicide-focused passive sensing is an active area with translational promise, but that rigorous prediction remains underrepresented relative to descriptive or in-sample analyses [19].

**Figure 1.**
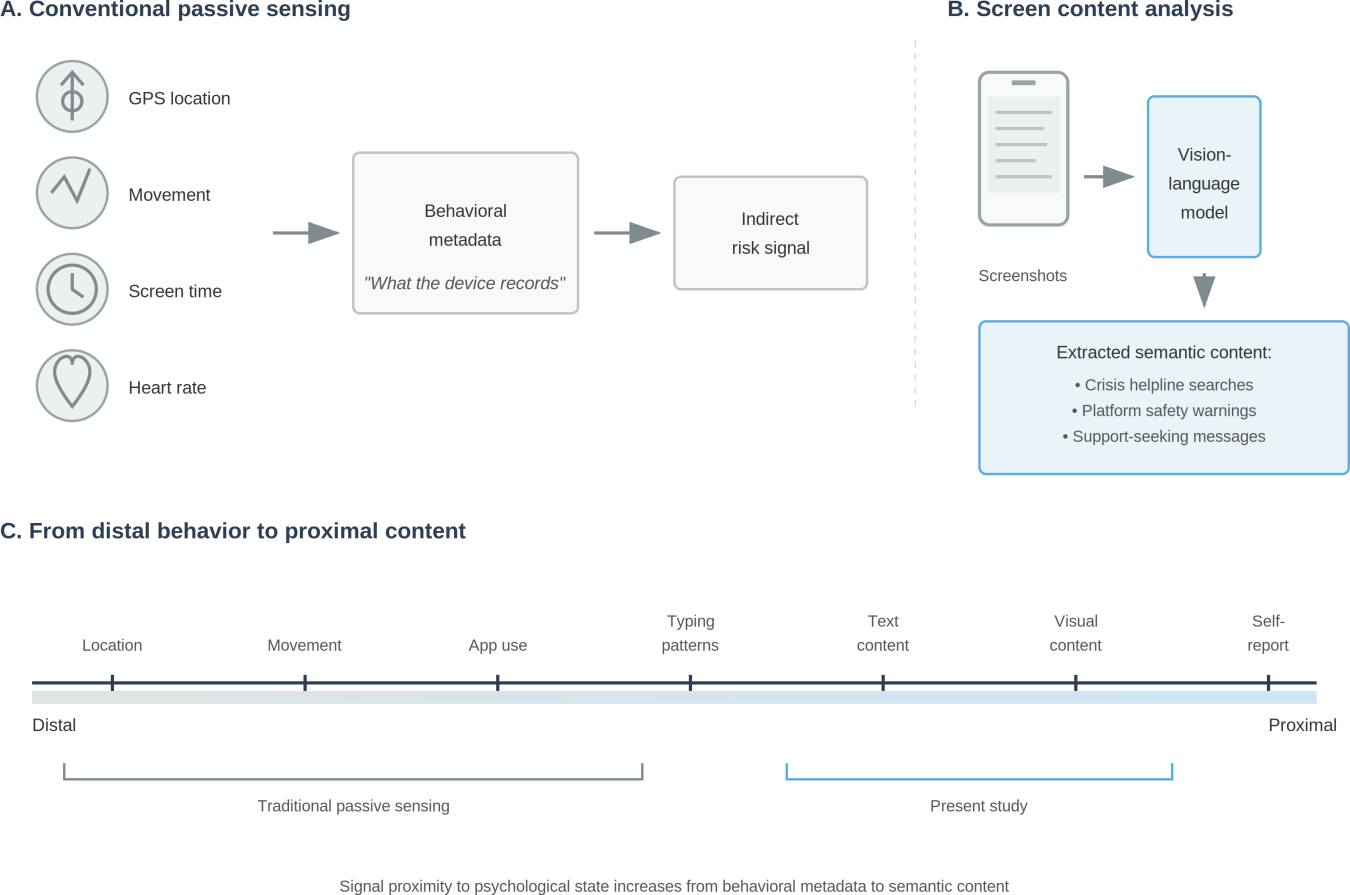
Conceptual overview of screen content analysis versus conventional passive sensing. (A) Conventional passive sensing captures behavioral metadata (GPS, movement, screen time, and heart rate). (B) Screen content analysis uses vision-language models to extract semantic content from screenshots. (C) Signal proximity to psychological state increases from distal behavioral metadata to proximal screen content.

A central challenge of many passive sensing approaches is that commonly used behavioral metadata (eg, GPA, screen time, and app counts) can be distal from the psychological mechanisms that researchers and clinicians need to understand and intervene upon to prevent suicide in real-time [7,20], meaning embedded in digital interactions, not only linguistic content, but also visual context, app interfaces, and media, as distinct from traditional passive sensing, which captures the mechanics of behavior (metadata, movement, and usage duration; see Figure 1A-1C). Importantly, semantic sensing exists on a continuum rather than being synonymous with any single data stream.

One increasingly used intermediate semantic modality is passively logged keyboard text input, which captures self-generated language (ie, what people type) during naturalistic smartphone use. Keyboard sensing has been used to extract clinically relevant linguistic markers from everyday communication (eg, pronoun use, sentiment, and other language features) and to link typed language and keystroke-related features to mental health symptoms over time [21-23]. Emerging work in adolescents also demonstrates the feasibility of detecting suicide-related language in passively collected keyboard entries using youth-tailored lexicons, highlighting the promise of semantic signals [24]. Although keyboard logging meaningfully moves beyond purely mechanical signals, it provides only a partial view of the person’s digital environments by reflecting primarily outgoing content and generally does not capture incoming messages, viewed text, or graphical/user interface context that may shape meaning.

In contrast, screenomics, referring to the high-frequency capture of smartphone screenshots every 5 seconds while in use, offers a richer semantic representation by incorporating incoming and outgoing communication, consumed content (ie, what a person reads/watches/is exposed to), and the visual and structural context of that content [25], aligned with a more fine-grained semantic sensing approach. Such access to broader digital environments may be especially important for detecting acute SI because risk-relevant signals may be contextual rather than explicitly self-disclosed [16-18]. We therefore conceptualize screenshots as capturing the broadest semantic substrate of mobile experiences while recognizing keyboard sensing as a narrower, yet meaningful, semantic channel within the passive sensing literature.

Several findings from the literature inform our approach to applying screenomics to the detection of suicide risk. Temporal proximity matters; suicide-related outcomes are most predictable over short windows [2,9]. Integrating modalities also tends to help, though gains from adding active self-report to passive signals [3,15] may partly reflect shared method variance when EMA items serve as both input and outcome [26]. We therefore focus on purely passive prediction to isolate the unique contribution of semantic information embedded in everyday digital environments.

Evaluation strategy poses an additional challenge in semantic sensing and digital phenotyping. Digital behaviors have idiosyncratic meanings; between-person patterns need not reflect within-person dynamics [5,6,27], as evidenced by the common finding that within-person (temporal holdout) prediction performance exceeds between-person (subject holdout) prediction [28]. This presents a critical evaluation challenge. While person-specific baselines may offer superior predictive power, the intended clinical use case determines the appropriate evaluation strategy. Models intended for monitoring individuals over time require temporal holdouts within a person to assess temporal generalization, for example, a calibration period in a JITAI [29] that learns participant-specific behavior patterns before implementing interventions in a testing phase. In contrast, models intended for screening new patients require person-level splits to assess generalization across individuals [30,31]. Critically, both approaches require evaluation strategies aligned with their intended use case; for instance, performance estimates can be substantially inflated when temporal and between-person sources of variance are conflated within train/test splits [19,32].

### Current Study

We test whether high-resolution smartphone screenshots, capturing what people actually read, watch, and type, can support moment-to-moment prediction of SI, which will ultimately support JITAI-style deployment. We leverage open-source foundation models (eg, Qwen2.5-VL [Alibaba Cloud], LFM2-VL [Liquid AI], and Qwen3 [Alibaba Cloud]) for images and text to learn representations directly from screenshot pixels and extracted text, rather than hand-crafted features. We compare 2 approaches, vision-language models (VLMs) that process screenshots as images, capturing layout, app context, and visual content, versus text-only models that operate on Optical Character Recognition (OCR)-extracted text alone. To align the signal with clinical decision points, we target the 2 hours preceding each EMA, a window designed to capture rapid risk fluctuations that are actionable, in real time.

Because clinical deployment faces 2 distinct generalization problems, we frame evaluation around 2 use cases. First, within-person risk detection aims to learn a person’s baseline and detect departures from it, ultimately serving to support personalized JITAIs. Second, across-subject generalization asks whether models trained on one cohort can provide useful risk estimates for entirely new individuals, as found in screening approaches. The former prioritizes temporal transfer within a person; the latter emphasizes robustness across heterogeneous digital habits. We also compare granularity—single-screenshot predictions versus brief, EMA-level summaries—testing whether short-horizon temporal context improves performance.

## Methods

### Sample

The sample consisted of 79 adults who reported past-month suicidal thoughts or behaviors, owned an Android-based smartphone, and demonstrated variability on the variables of interest during 28 days of data collection. Of the 79 enrolled participants, 70 were retained in the analytic sample, 3 were excluded due to no screenshots captured, and 6 due to complete nonresponse to EMA assessments. Participants were recruited via social media advertisements and community flyers from a midsized city in the Midwest. Participants ranged in age from 20 to 63 years (mean 35.15, SD 11.07); 68.% (54/79) were female; 84.8% (67/79) identified as White, 6.3% (5/79) as Black, 6.3% (5/79) as American Indian/Alaska Native, and 2.5% (2/79) as another race; and 92.4% (73/79) identified as non-Hispanic/Latino. Most participants (83.8%; 66/79) met diagnostic criteria for at least one psychiatric disorder, and 64.9% (51/79) met current diagnostic criteria for 2 or more disorders (mean 2.55, SD 1.86). Further, 72.2% (57/79) had a lifetime history of a suicide plan (38% (30/79) in the past year), and 64.6% (51/79) had a lifetime history of a suicide attempt (12.7% (10/79) in the past year). Two participants self-presented for psychiatric hospitalization for suicidal crises during the study period; no participants reported engaging in a suicide attempt.

Screenshots were automatically captured every 5 seconds during active phone use via the ScreenLife Capture app (University of Washington) [33], with modifications made by the study team. The study captured 7,501,670 screenshots in total, with participants averaging 92,613 screenshots each (median 85,795; IQR 20,469-130,888) across the study period. During the informed consent process, participants were fully informed of all data collection procedures (ie, screenshot capture every 5 seconds their phone was in use), including the possibility of capture of sensitive data throughout the study period, data storage procedures, and steps taken to help protect participant confidentiality (ie, limited viewing of raw screenshot files). Limits of confidentiality were also discussed, including circumstances during which the research staff was concerned for their immediate safety. All participants received resources at study enrollment. If nonzero active SI was reported via EMA, an automated pop-up of crisis resources was provided. If high levels of active SI (ie, >4 out of 5) were reported, the study team reached out to conduct a comprehensive risk assessment.

### Ecological Momentary Assessment

Participants received 6 signal-contingent EMA prompts per day (ie, randomized within 2-hour windows across a 12-hour block) via the LifeData (LifeData LLC) app, each taking 3‐4 minutes to complete. The overall EMA compliance rate was 68.8%, with an average of 3.4 hours elapsed between EMA surveys. Two questions assessed momentary (ie, “At this moment…”), active SI (ie, “I think about taking my life;” “I want to die”), which have been previously validated [34]. These items were answered on a 5-point Likert scale (1=not at all to 5=very much) and summed to create a composite score (range: 2‐10). The continuous SI composite exhibited pronounced floor effects, with 63%‐69% of EMAs having scores of 2 (both items at minimum), and only 10%‐15% scoring 5 or above (indicating at least moderate endorsement on one item). We therefore dichotomized the outcome as 0=no SI (composite =2) and 1=SI present (composite >2; positive class for AUPRC). In the temporal holdout split, SI was present in 36.5% of train EMAs (1086/2974) and 33.9% of test EMAs (381/1123). In the subject holdout split, SI was present in 40.0% of train EMAs (871/2177) and 31.0% of test EMAs (582/1880).

### Data Subsetting

For the purposes of this study, we removed screenshots that occurred more than 2 hours prior to the EMA assessment. The resultant dataset comprised 2,554,692 screenshot observations collected from 70 unique participants over the 28-day study period, with substantial variability in data contribution across individuals (mean 36,495.60 screenshots per participant, SD 26,999.18; range 7‐129,680). Compliance was 68.11% for active SI, and text extracted from screenshots was present in 99.94% of observations (2,553,240 rows), with only 1452 instances containing no detectable text. The extracted text varied considerably in length (mean 224.13 characters, SD=194.26; range=0‐2491), reflecting the diverse nature of smartphone screen content captured during naturalistic use, from brief notifications to lengthy articles or messages. All extracted text was retained without filtering, given the models’ ability to handle variable input lengths and the challenge of defining noise in naturalistic smartphone data.

### Performance Evaluation

Our intended applications are (1) within-person personalized risk detection and (2) across-subject generalization. These require different evaluation strategies [30-32]: temporal holdouts assess whether person-specific patterns persist over time (relevant for personalized monitoring), while subject holdouts assess generalization to new individuals (relevant for screening). Performance estimates can be inflated by 100%‐300% when the evaluation strategy is misaligned with the intended use case [35,36]. We report only out-of-sample predictions under both strategies.

### Train/Test Partitioning

For temporal holdout validation, we trained on the first 70% of each participant’s EMA assessments and tested on the final 30%, simulating prospective deployment and avoiding interpolation across randomly intermixed time points [30,37]. For subject holdout validation, we performed a 50/50 split by participant ID, keeping all observations from a given person in a single fold to test performance on entirely new individuals and to respect ergodicity concerns [38,39]. More elaborate schemes (eg, blocked CV with gaps, leave-one-subject-out, constrained repeated random sampling-cross validation, and forward-chaining) are valuable [40-43] but were computationally prohibitive with foundation models.

### Prediction Targets, Labeling, and Aggregation

**Figure 2.**
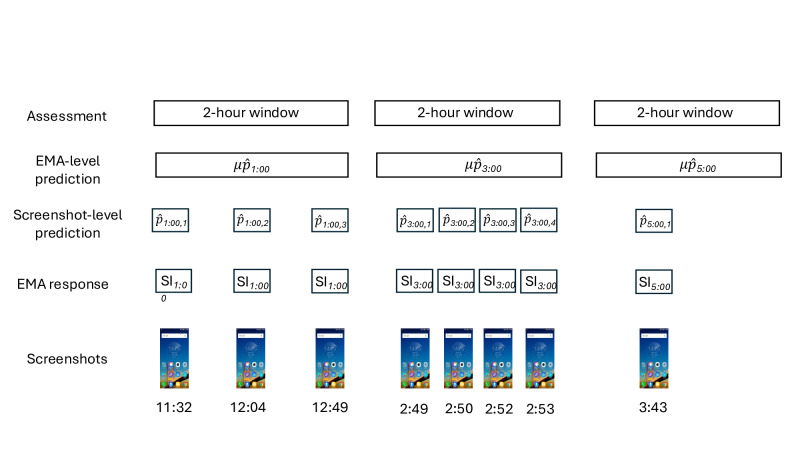
Data structure and prediction aggregation. Screenshots within the 2-hour window preceding each ecological momentary assessment (EMA) inherit that assessment’s suicidal ideation label. For example, screenshots captured at 11:32, 12:04, and 12:49 are labeled with the 1:00 PM EMA response (SI₁:₀₀) and aggregated to predict that assessment. Screenshot-level predictions (p̂) are generated for each image; EMA-level predictions (μp̂) aggregate these via summary statistics. EMA: ecological momentary assessment.

To couple signals to clinically actionable horizons, we restricted inputs to screenshots captured within the 2-hour window prior to each EMA [2,44]. Screenshots in this window inherited the label from the immediately following EMA. That is, for an EMA at time “t*”*, all screenshots from (*t*−2h, *t*) were labeled with that EMA’s SI response. Screenshots occurring after an EMA were never used to predict that assessment, ensuring strictly prospective prediction (see Figure 2 for an overview of the data design). We evaluated two granularities: (1) screenshot-level predictions and (2) EMA-level predictions that aggregate information across screenshots in a window. For EMA-level models, we computed summary features commonly used in short-horizon risk detection (eg, mean, max, SD, range, 75th-90th percentiles, proportion≥5 and ≥7 risk, skewness, and kurtosis). This design tests whether brief temporal context improves discrimination relative to single-frame decisions while maintaining strict separation between training and test periods/participants. Of note, we tested models that aggregated the data prior to training (eg, concatenating the text; subsampling images); however, none of these models evidenced an above-average change in predictive performance, likely due to limitations with the token input constraints.

These choices reflect a theoretical stance as much as a technical one: temporal holdouts split the probe to see whether person-specific digital patterns persist over time and are therefore suitable for personalized JITAIs; subject holdouts ask whether learned indicators transfer across heterogeneous digital habits—a harder, but necessary, condition for broader screening [31,39]. By using deployment-aligned evaluation strategies, we aim to provide performance estimates that are credible for real-world deployment in digital medicine [30,32].

### Evaluation Metrics

We report area under the receiver operating characteristic curve (AUC) and area under the precision-recall curve (AUPRC) as threshold-independent measures of discrimination on held-out data; AUPRC is especially informative under class imbalance because it emphasizes performance on the positive class [45-47]. Because our goal was to assess ranking ability rather than commit to an operational alert threshold, we did not report single-threshold metrics (eg, precision, recall, *F*_1_-score, and accuracy), which depend on a chosen decision rule and deployment prevalence [46,47].

To account for clustering of observations within participants, we computed 95% CIs using participant-level bootstrap resampling (1000 iterations). In each iteration, participants were sampled with replacement, and all observations from each sampled participant were included. AUC was computed on the resampled data, and percentile-based confidence intervals were derived from the bootstrap distribution. For model comparisons, we computed ΔAUC on paired bootstrap samples to obtain confidence intervals for the difference in discrimination between models.

Because pooled AUC computed across all participants can conflate within-person predictive accuracy with between-person differences in baseline risk [31,39], we conducted supplemental analyses to decompose these sources of discrimination. First, we computed person-level AUC for each participant who had both SI-present and SI-absent EMAs in the temporal holdout test set and reported the distribution of these values. Second, we assessed the contribution of between-person discrimination by replacing each observation’s predicted probability with that participant’s mean predicted probability across all test EMAs (effectively reducing predictions to a person-specific intercept) and computing AUC on these intercept-only predictions. The difference between the full and between-person-only AUC indexes the incremental contribution of within-person temporal variation in predictions.

### Text and Visual Feature Extraction

EasyOCR (Jaided AI [48]), a deep learning-based optical character recognition system, was applied to each screenshot to extract text content. The Python (Python Software Foundation) implementation of EasyOCR uses a combination of text detection and recognition models to identify and transcribe text regions from images. Across all extracted text blocks, EasyOCR confidence scores were highly variable (median 0.66, IQR 0.27‐0.93), reflecting heterogeneity in extraction quality across screenshot types. During manual inspection, we observed that text blocks with lower OCR confidence scores often contained inserted special characters rather than complete misreadings, making dictionary matching possible regardless of confidence thresholds. Therefore, we chose not to exclude any text blocks based on EasyOCR confidence scores.

Additionally, we applied Florence-2 (base version) [49], a VLM pretrained on diverse visual and textual tasks. For each screenshot, the system performed five parallel extraction tasks: (1) optical character recognition to extract all visible text content, (2) detailed visual captioning to describe screen elements and layout, (3) condensed content analysis, (4) application identification through direct model querying (“Which mobile application is shown in this screenshot?”), and (5) social engagement feature detection with interaction cues, call-to-action elements, and engagement features. Application identification combined both heuristic text matching against extracted OCR content (searching for app-specific keywords like “Facebook,” “Instagram,” and “WhatsApp”) and the model’s direct predictions.

### Zero-Shot Learning Analysis

To evaluate prediction without person-specific calibration, we applied zero-shot models that used no training data from this sample, relying entirely on pretrained weights. Risk scores were extracted from Llama 3.2 11B Vision-Instruct (Meta Inc; see Multimedia Appendix 1) using regular expression pattern matching to identify numeric values (0‐10) at the end of each response. Missing EMA scores (n=3543) were excluded from analysis. Extraction failures were rare (0.05% of observations), did not predict SI (AUC=0.50), and results were robust to imputation strategy. Additionally, to evaluate zero-shot transferability of existing mental health–specific models, we applied Mental-FLAN-T5-XXL [50], an 11-billion parameter model instruction-finetuned on multiple Reddit (Reddit Inc)-based mental health datasets, including suicide ideation classification (SDCNL), depression severity (DepSeverity), and stress detection (Dreaddit). This model was selected because it previously outperformed GPT-3.5 and GPT-4 on in-domain mental health classification tasks, representing a strong prior for text-based risk assessment. We used a classification-based prompt asking the model to categorize risk as “none, low, moderate, high, or severe” based on the OCR-extracted text; categorical outputs were mapped to ordinal probability scores (0.0‐1.0) for analysis.

### Fine-Tuning

All models were trained to predict a binary indicator of active SI (composite EMA score >2) using binary cross-entropy loss with logits. No class weighting or resampling was applied; instead, performance was evaluated using threshold-independent metrics (AUC and AUPRC), which are robust to class imbalance. All models were implemented in PyTorch (Meta Inc) using HuggingFace Transformers (Hugging Face) and trained on NVIDIA L40S GPUs (48GB VRAM; NVIDIA Corp) using distributed data parallelism where applicable. Complete prompts are provided in Multimedia Appendix 1.

### Qwen2.5-VL (Vision-Language Model)

Qwen2.5-VL adds native dynamic-resolution processing and absolute time encoding for long-video understanding while preserving strong text generation. A redesigned ViT with windowed attention retains native image resolution at lower compute. The series is released in 3B, 7B, and 72B variants; the 72B model performs on par with leading multimodal systems on document/diagram tasks [51]. We used the 3B and 7B variants.

The model was fine-tuned using the Qwen2.5-VL-3B-Instruct base model with Low-Rank Adaptation (LoRA) [52] to efficiently adapt the VLM for suicide-risk prediction. The LoRA configuration used a rank (r) of 16 with a scaling factor (α) of 32, targeting the query, key, value, and output projection layers (q_proj, v_proj, k_proj, o_proj) of the attention mechanism. Training was conducted for 3 epochs with a batch size of 2, using the AdamW optimizer with a learning rate of 1e-5 and weight decay of 0.01. Gradient clipping was applied with a maximum norm of 0.5 to ensure stable training. Input images were resized to 224×336 pixels.

### Qwen3 (Large Language Model Series)

Qwen3 comprises dense and mixture-of-experts large language models from 0.6B to 235B parameters. A unified design supports both a reasoning (“thinking”) mode and a fast conversational (“non-thinking”) mode, with a user-controllable “thinking budget” to trade off latency and accuracy. Qwen3 reports state-of-the-art results across coding, math, and agent tasks; expanded coverage of 119 languages; and Apache-2.0 licensing [53]. We used the 1.7B and 4B variants. For zero-shot learning only, we applied the 30B-A3B variant (using a different prompt; see Multimedia Appendix 1).

The text-based suicide risk prediction model used Qwen3-1.7B as the base language model, fine-tuned with LoRA targeting a comprehensive set of attention and feed-forward network modules (q_proj, v_proj, k_proj, o_proj, gate_proj, up_proj, down_proj). The LoRA configuration used a rank of 16 with an alpha scaling factor of 32 and dropout rate of 0.1. Training was conducted for up to 10 epochs with an effective batch size of 8 (batch size of 2 with gradient accumulation over 4 steps), using the AdamW optimizer with a learning rate of 2e-6, weight decay of 0.01, and gradient clipping at 0.5. The model used mixed precision training with bfloat16 and implemented a linear learning rate schedule with 10% warmup steps. Text inputs were tokenized with a maximum sequence length of 2048 tokens and early stopping based on EMA-level AUC performance (patience of 5 epochs, minimum delta of 0.001).

### Liquid AI Foundation Models (LFM2)

LFM2 are compact, on-device large language models using a hybrid architecture (10 multiplicative-gated convolutional blocks plus 6 grouped-query attention blocks discovered via STAR NAS), trained on ~10T tokens with knowledge distillation. Released at 350M, 700M, and 1.2B parameters. On automated benchmarks, the 350M model is competitive with Qwen3-0.6B and Llama-3.2-1B, while the 1.2B model approaches Qwen3-1.7B [54].

### Liquid AI LFM2-VL (Vision-Language Models)

LFM2-VL extends LFM2 with a SigLIP-2 NaFlex vision encoder and a lightweight projector, offering 2 open-weight variants, 450M and 1.6B parameters. Models process images at native resolution up to 512×512 with patching and an optional thumbnail for global context and report up to 2×faster GPU inference than similarly sized VLMs. Reported results include RealWorldQA ≈52.3% for the 450M model and ≈65.2% for the 1.6B model, competitive within the ~0.5–2B class [55,56].

The image-based suicide risk prediction model was fine-tuned using the LFM2-VL-450M vision-language foundation model as the base architecture. Fine-tuning was performed using the Transformer Reinforcement Learning Supervised Fine-Tuning framework with LoRA applied to enhance parameter efficiency. The LoRA configuration used a rank (r) of 16 and scaling factor (alpha) of 32, targeting attention and feed-forward modules, including q_proj, k_proj, v_proj, o_proj, fc1, fc2, gate_proj, up_proj, and down_proj. Training was conducted for 3 epochs with a batch size of 4 and gradient accumulation over 4 steps (effective batch=16), using the AdamW optimizer with a learning rate of 2×10⁻⁵, weight decay of 0.01, and a cosine learning-rate scheduler with 10% warm-up. Training used bfloat16 mixed precision with TF32 enabled and applied gradient clipping (max norm=0.5) to ensure numerical stability. Input images were dynamically resized to 224×336 pixels, and training/validation splits followed a 50/50 and 70/30 data division. Model evaluation was based on accuracy and AUC, computed on a held-out validation subset. All fine-tuning and evaluation were executed on a single-GPU SLURM node using efficient on-the-fly image loading.

The text-only suicide risk prediction model was trained on a large-scale dataset using the LFM-2 Text framework. The configuration used *r*=64, alpha=128, and dropout=0.05, targeting key attention and MLP projection modules (q_proj, k_proj, v_proj, o_proj, gate_proj, up_proj, down_proj). Training was performed for 1 epoch on the full dataset without intermediate evaluations to maximize throughput, using the AdamW optimizer with a learning rate of 2×10⁻⁶, weight decay of 0.01, and gradient accumulation steps of 4 (effective batch=8). Mixed precision training with bfloat16 and TF32 acceleration was used throughout. The model incorporated gradient checkpointing for memory efficiency and used a cosine learning-rate schedule with a warm-up ratio of 0.1. Text inputs were tokenized with a maximum sequence length of 2048 tokens, and final model evaluation was conducted posttraining using a separate validation pipeline. Training used bits-and-bytes quantization (4-bit or 8-bit configurable) for efficient large-scale optimization.

### Secondary Analyses

#### Temporal and Behavioral Confounding

To assess the extent to which prediction could be driven by temporal or behavioral regularities rather than semantic content, we trained gradient-boosted decision tree baselines using XGBoost (XGBClassifier) [57]. These models served as nonsemantic baselines for (1) SI prediction using temporal/behavioral features and (2) EMA missingness prediction. All XGBoost models used 100 trees, a maximum depth of 5, a learning rate of 0.1, histogram-based tree construction (tree_method=hist), and log loss as the objective function, with no class weighting applied. Features for the confounding baseline included cyclical encodings of hour-of-day and day-of-week (sine and cosine), a weekend indicator, and the number of screenshots within each EMA window. The missingness baseline additionally included coarse time-of-day categories and study day index. Models were trained on the full training split in a single fit (no epochs or early stopping) with a fixed random seed (42), using GPU acceleration when available and CPU otherwise. These baselines provide a lower-bound comparison for semantic models by capturing timing and usage intensity without access to screen content.

#### Lexical Screening for Between-Person Prediction

Initial experiments established baselines using random selection and evenly spaced temporal sampling of 30 screenshots per 120-minute window preceding each assessment (AUC=0.599). We then implemented dictionary-based selection using a 276-term crisis [16] dictionary across seven categories (suicidal thoughts, nonsubstance methods, substance use, sleep, help-seeking, hopelessness, and general risk), prioritizing screenshots with the highest crisis term counts (AUC=0.615, AUPRC=0.452 vs 0.310 baseline). Enhanced approaches incorporating temporal weighting (recent screenshots weighted 1.0 declining to 0.5), context windows (100 characters around crisis terms), and similarity-based diversity enforcement (70% threshold) reduced performance (AUC=0.597). Finally, we developed a data-driven approach that learned predictive terms directly from screenshot-level TF-IDF features using logistic regression on individual screenshots labeled by their associated SI scores, then used the discovered terms’ coefficients to score and select screenshots for final EMA-level prediction, though all methods showed substantial overfitting (train-test AUC gaps of 0.33‐0.41), suggesting the need for stronger regularization or alternative architectures.

#### EMA Missingness Sensitivity Analyses

Because analyses relied on screenshots linked to completed EMAs (~69% compliance), we conducted sensitivity analyses to assess whether non-response could systematically bias performance estimates. First, to evaluate whether missingness is predictable from the same features used in SI prediction, we trained models to predict EMA completion rather than SI. An XGBoost baseline used temporal and behavioral features (cyclical encodings of hour-of-day and day-of-week, weekend indicator, screenshot count per EMA window, coarse time-of-day categories, and study day index). We then trained a separate VLM (Qwen2.5-VL) to predict EMA completion from screenshot content, using the same fine-tuning procedure described above, to assess whether screen content additionally predicted missingness. Second, to directly test whether the SI prediction model produced systematically different risk estimates for completed versus missed EMAs, we applied the trained SI temporal holdout model (Qwen2.5-VL) to all screenshots in the test set, regardless of whether the corresponding EMA was completed. Predictions were aggregated to the EMA level (mean) and compared between completed and missed EMAs using a mixed effects model (predicted risk~completion status, random intercept for participant) and within-person effect sizes (person-centered Cohen *d*). Phone use intensity (screenshot count per 2-hour window) was compared using the same specification.

### Interpretation

We first identified the top 1000 model-predicted probability screenshots from the Qwen-VL 70/30 model and passed each image through a Qwen-VL analysis script that applied a fixed, suicide-relevant prompt to produce structured natural-language descriptions of the on-screen content (platform/app, interaction type, safety dialogs, crisis/help-seeking language, and contextual cues; see Multimedia Appendix 1); these descriptions comprised the text corpus for interpretation. After text cleaning (lowercasing, boilerplate removal, and retention of platform and suicide-relevant terms), we vectorized the corpus and ran a sweep of topic models, Latent Dirichlet Allocation (LDA) [58] on count features and nonnegative matrix factorization (NMF) on TF-IDF features, across candidate solutions (k ≈ 5‐15) to recover a compact, coherent set of suicide-relevant digital contexts. Each topic from the LDA and NMF runs was then graded into one of 4 salience bands—high (explicit crisis/help/safety), medium (operationally adjacent screens, such as platform safety systems or multi-app assessments), support (coping/help content), and subtle (ambient/contextual screens with weaker signal)—using the top terms and exemplar descriptions. Finally, we rendered smartphone-style synthetic mockups for each topic using DALL-E 3, instantiating that topic’s key user interface elements (eg, an Instagram [Meta Corp] post with a self-harm warning, a crisis chat thread, or an assessment-like mobile card) so VLM-derived topics could be visually inspected for interpretability. These mockups are illustrative visualizations of content categories and do not depict actual participant screenshots; topic labels should be interpreted as general thematic clusters rather than literal content descriptions.

### Ethical Considerations

This study was approved by the institutional review board at the University of Notre Dame (#21-12-6965) and the University of Wisconsin–Madison (#2024-1031). All procedures were conducted in accordance with the Declaration of Helsinki. During the informed consent process, participants were fully informed of all data collection procedures, including screenshot capture every 5 seconds during active phone use, the possibility of capturing sensitive data, data storage procedures, and steps taken to protect participant confidentiality (ie, limited viewing of raw screenshot files). Limits of confidentiality were also discussed, including circumstances in which the research team was concerned for a participant's immediate safety. All participants received crisis resources at enrollment; if nonzero active SI was reported via ecological momentary assessment, an automated pop-up of crisis resources was provided, and if high levels of active SI (ie, ≥4 out of 5) were reported, the study team conducted a comprehensive risk assessment. To protect participant privacy, raw screenshot data are not publicly available, and all model training and evaluation were conducted within a Health Insurance Portability and Accountability Act (HIPAA)–secure computing environment. Synthetic images used for interpretation were generated with no real participant data, and all were labeled “SYNTHETIC–RESEARCH ONLY.” Participants were compensated up to $230 for their involvement: $40 for the baseline session, $100 for ecological momentary assessment completion, a $35 incentive bonus for completing at least 75% of ecological momentary assessments, and $55 for the screenshot capture period.

## Results

### Overview

The AUC and AUPRC values across models and data types are summarized in Table 1.

**Table 1.** Model performance across evaluation conditions. Area under the receiver operating characteristic curve (AUC) and area under the precision-recall curve (AUPRC) for vision-language (images) and text-only (text) models under temporal holdout (70/30) and subject holdout (50/50) evaluation. Positive class (suicidal ideation [SI] present) prevalence: 33.9% in temporal holdout test and 31.0% in the subject holdout test; baseline AUPRC equals prevalence. 95% CIs in brackets computed via participant-level bootstrap (1000 iterations).

Model	Level	Temporal holdout		Subject holdout	
		AUC^a^ (95% CI)	AUPRC^b^ (95% CI)	AUC (95% CI)	AUPRC (95% CI)
Images					
Qwen	Screenshot	0.747 (0.671-0.813)	0.659 (0.506-0.773)	0.517 (0.500-0.584)	0.382 (0.249-0.499)
Qwen	EMA^c^	0.830 (0.743-0.904)	0.767 (0.580-0.892)	0.498 (0.504-0.680)	0.355 (0.202-0.435)
LFM2^d^	Screenshot	0.778 (0.692-0.842)	0.695 (0.509-0.800)	0.530 (0.491-0.568)	0.310 (0.225-0.471)
LFM2	EMA	0.806 (0.690-0.881)	0.752 (0.589-0.860)	0.511 (0.490-0.620)	0.312 (0.202-0.429)
Text					
Qwen	Screenshot	0.683 (0.616-0.739)	0.586 (0.418-0.745)	0.524^e^ (0.500-0.605)	0.283^e^ (0.248-0.595)
Qwen	EMA	0.793 (0.791-0.859)	0.718 (0.507-0.816)	0.563^e^ (0.488-0.697)	0.312^e^ (0.234-0.582)
LFM2	Screenshot	0.488 (0.471-0.505)	0.315 (0.218-0.416)	0.521 (0.497-0.589)	0.390 (0.252-0.597)
LFM2	EMA	0.580 (0.606-0.652)	0.306 (0.202-0.435)	0.550 (0.488-0.697)	0.380 (0.235-0.583)

a AUC: area under the receiver operating characteristic curve.

b AUPRC: area under the precision-recall curve.

c EMA: ecological momentary assessments.

d LFM2: Liquid AI Foundation Model.

e Models using high-level Florence-2 features rather than raw OCR text.

### Temporal Holdout Evaluation

#### Image

Using Qwen2.5-VL, at the screenshot level, the model evidenced an AUC of 0.747 and an AUPRC of 0.659. At the EMA level, there were similar results across several summary statistics, with the mean performing among the best, with an AUC of 0.830 and AUPRC of 0.767, substantially exceeding the baseline of 0.339 (positive class prevalence). Not much was lost with the application of the LFM2-VL model, with an AUC of 0.778 and AUPRC of 0.695 at the screenshot level and an AUC of 0.806 and AUPRC of 0.752 using the mean at the EMA level.

#### Text

Using Qwen3, performance was somewhat attenuated relative to the image-based models but still showed meaningful discrimination. At the screenshot level, the model achieved an AUC of 0.683 and an AUPRC of 0.586. Aggregating to the EMA level improved performance, mirroring the pattern seen in the image analyses: the EMA-level summary reached an AUC of 0.793 and an AUPRC of 0.718. Somewhat surprisingly, LFM2 performed markedly worse, with screenshot-level results around chance, while the aggregated metrics were still far below the VL results.

#### Comparison

Image-based models showed a small advantage over text-based models. At the EMA level, the Qwen image model (AUC=0.83, 95% CI 0.74-0.90) outperformed the Qwen text model (AUC=0.79, 95% CI 0.70-0.86), with a difference of ΔAUC=0.04 (95% CI 0.003-0.07). Comparing vision-language architectures, LFM2 performed comparably to Qwen (AUC=0.80, 95% CI 0.71-0.89; ΔAUC=0.02), supporting feasible on-device deployment without substantial performance loss. All confidence intervals were computed using participant-level bootstrap (1000 iterations) to account for clustering within individuals.

#### Calibration

**Figure 3.**
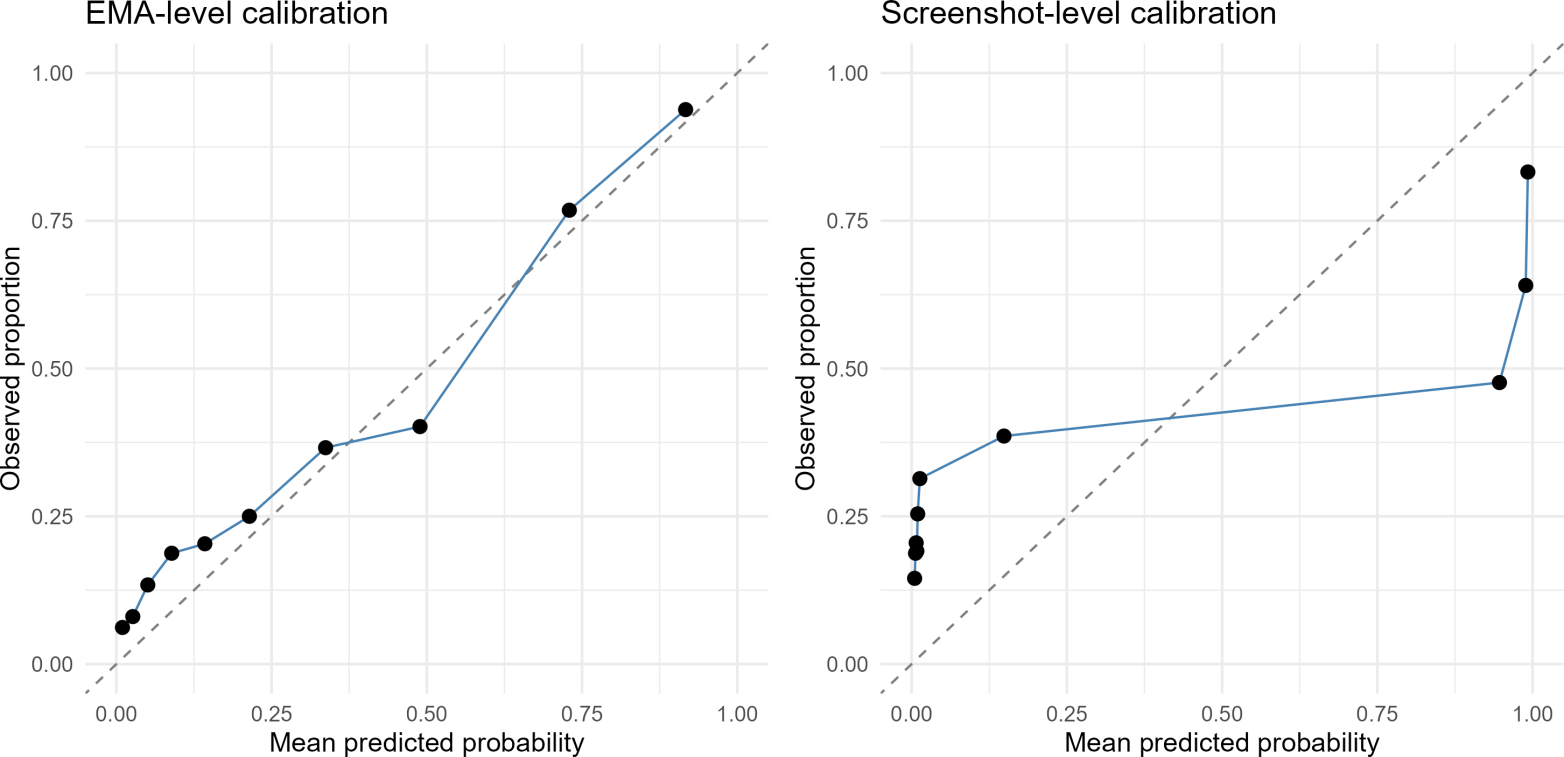
Calibration curves for the best-performing model (Qwen2.5-VL, temporal holdout) at ecological momentary assessment (EMA) level (left) and screenshot level (right). Each point represents one decile bin of predicted probabilities; the dashed diagonal indicates perfect calibration. At the EMA level, predictions are monotonically related to observed suicidal ideation rates but exhibit systematic underconfidence (calibration slope=4.89), with low-risk predictions slightly overestimating and high-risk predictions substantially underestimating observed event rates. At the screenshot level, predicted probabilities are heavily compressed: approximately 80% of observations fall below 0.15, with a small proportion near 1.0, illustrating the probability compression that EMA-level aggregation partially corrects. EMA: ecological momentary assessment.

For the best-performing model (Qwen image, temporal holdout), calibration was assessed at the EMA level (see Figure 3). The calibration slope was 4.89 (ideal=1), indicating substantial underconfidence. Predicted probabilities were compressed toward the center relative to observed outcomes. The calibration intercept was −2.24 (ideal=0), and the Brier score was 0.147. At the screenshot level, the calibration slope was closer to unity (1.90), but overall accuracy was lower (Brier=0.258), reflecting noisier frame-level predictions that EMA-level aggregation sharpens at the cost of increased probability compression. We evaluated whether post-hoc Platt scaling could correct this miscalibration by fitting a logistic recalibration model on a held-out calibration subset (n=523 EMAs) and applying it to an evaluation subset (n=600 EMAs). Platt scaling did not substantially improve calibration (postrecalibration slope=4.74, Brier=0.146), suggesting that the probability compression induced by aggregating screenshot-level predictions to EMA-level summaries requires more sophisticated calibration approaches than simple monotonic transformations.

### Subject Holdout Evaluation

#### Image

Compared to the temporal holdout models, the subject holdout models showed clear attenuation in performance. Using Qwen2.5-VL on image features at the screenshot level, the model achieved an AUC of 0.517 and an AUPRC of 0.382. Aggregating to the EMA level did not improve performance (AUC=0.498; AUPRC=0.355), suggesting that in the between-person setting, there was limited additional discriminative signal to recover from averaging across a person’s screenshots. AUPRC values (0.31‐0.39) were near the baseline prevalence of 0.31, indicating minimal discrimination beyond chance. For the LFM2-VL image models, performance can be considered at chance.

#### Text

A similar pattern was observed for text-only features. At the screenshot level, the Qwen text model produced an AUC of 0.524 and an AUPRC of 0.283, indicating only marginal separation. EMA-level aggregation improved things slightly (AUC=0.563; AUPRC=0.312), but performance still lagged the temporal holdout evaluations. Of note, this performance was based on application to the high-level text features, not raw text (AUC=0.467; AUPRC=0.354). As with the image models, the LFM2-VL text models in this between-person context performed similarly, but with higher AUPRC values (0.390 and 0.380) and were trained on the raw text.

### Zero-Shot Learning

At the image level, LLaMA risk scores demonstrated minimal predictive ability (AUC=0.511; AUPRC=0.410). At the EMA level, the mean risk score across images within an EMA showed the strongest predictive ability (AUC=0.578; AUPRC=0.436), followed by SD (AUC=0.568; AUPRC=0.427). Zero-shot models were evaluated without any training or fine-tuning on data from this study, relying entirely on pretrained weights. Their weak performance likely reflects the absence of individual-specific calibration, which limits the model’s ability to learn person-specific baselines and idiosyncratic digital behavior patterns.

Applied only to the 50/50 test set, we applied Qwen3-30B-A3B to both the text and images. For the text, at the screenshot level, the AUC was 0.541 and AUPRC of 0.343, and at the EMA level (mean), the AUC was 0.583 and AUPRC of 0.385. In images, the screenshot AUC was 0.518 with an AUPRC of 0.329, and at the EMA level (mean), the AUC was 0.552 and the AUPRC was 0.398.

Performance of Mental-FLAN-T5—despite its strong prior results on social media text—was limited on screenshot OCR; at the screenshot level, discrimination was near chance (AUC=0.504; AUPRC=0.322). Aggregating to the EMA level using mean scores yielded modest improvement (AUC=0.558; AUPRC=0.360), suggesting that even specialized mental health models do not transfer effectively to passively collected screenshot content.

### Sensitivity and Secondary Analyses

#### Within-Person Versus Between-Person Discrimination

To assess the extent to which pooled performance reflects within-person temporal discrimination versus between-person differences in baseline risk, we computed person-level AUCs for participants with outcome variability in the temporal holdout test set (reported here for the best-performing model, Qwen2.5-VL with EMA-level mean aggregation). Each participant contributed a median of 13 test EMAs (IQR 7‐26; range 1‐48). Of 64 participants, 19 had all-zero test outcomes and 6 had all-positive, reflecting genuine individual differences in SI base rates given the reasonably sized per-person test sets, leaving 39 out of 64 (61%) with computable person-level AUCs. The median person-level AUC was 0.70 (mean 0.70, SD 0.13; IQR 0.60‐0.80), indicating that the model discriminated higher- from lower-risk time points within individuals above chance. A between-person-only analysis that replaced each observation’s prediction with the participant’s mean predicted probability yielded an AUC of 0.86, indicating that a substantial portion of the pooled AUC (0.83) reflects the model’s calibration to person-specific base rates. This decomposition is consistent with the intended deployment scenario: a calibration period allows the model to learn individual baselines, while temporal variation in predictions provides additional within-person discrimination for ongoing monitoring.

#### Temporal and Behavioral Confounding

To assess whether prediction was driven by temporal or behavioral regularities rather than semantic content, we compared fine-tuned VLMs against a non-semantic baseline using only temporal and usage features (cyclical encodings of hour-of-day and day-of-week, weekend indicator, and screenshot count per EMA window). The temporal baseline achieved near-chance discrimination (AUC=0.52; AUPRC=0.39 for temporal holdout; AUC=0.51; AUPRC=0.37 for subject holdout), with AUPRC values approximating class prevalence as expected for uninformative classifiers. In contrast, models processing screenshot content achieved substantially higher discrimination in temporal holdout: ΔAUC =+0.31; ΔAUPRC =+0.37 for Qwen image (EMA-level); ΔAUC =+0.28; ΔAUPRC =+0.36 for LFM2 image; and ΔAUC =+0.27; ΔAUPRC =+0.33 for Qwen text. The minimal improvements observed in subject holdout (ΔAUC≤0.05; ΔAUPRC near zero or negative) reflect the previously noted generalization challenge rather than confounding, as the temporal baseline performed equivalently poorly across both evaluation strategies.

#### Lexical Screening for Between-Person Prediction

Given the weak performance of the between-person models, formal comparisons across model architectures were not pursued; instead, we conducted exploratory follow-up analyses using substantially simplified approaches (see Multimedia Appendix 1). Specifically, we applied a dictionary-based screening procedure that used a 276-term crisis lexicon [16] spanning 7 categories (suicidal thoughts, nonsubstance methods, substance use, sleep, help-seeking, hopelessness, and general risk) to identify screenshots with the highest concentration of crisis-relevant terms. We then selected the 30 screenshots with the largest term counts and fit regularized logistic regression models to tf–idf representations of the extracted text. Performance was modest, yielding an AUC of 0.615 and an AUPRC of 0.452.

#### EMA Missingness

To assess whether missingness could bias performance estimates, we examined predictions from 2 angles. First, models trained to predict EMA completion (rather than SI) from the same temporal and behavioral features achieved modest discrimination (AUC=0.60); adding VLM-derived content features yielded only marginal improvement (AUC=0.66), indicating that missingness is only weakly predictable from the features used in SI prediction. Second, we applied the trained temporal holdout model (Qwen2.5-VL) to all screenshots in the test set, regardless of whether the corresponding EMA was completed. In the test set (completion rate=61.7%; 1111 of 1802 EMAs), predicted SI risk did not differ systematically between completed and missed EMAs (within-person Cohen *d*=0.03; mixed effects *β*=.005; *t*=0.60). Phone use intensity was modestly higher before completed EMAs (mean 444 vs 411 screenshots per 2-hour window; *β*=55.8; *t*=3.19), consistent with greater phone engagement preceding EMA response. However, conditional on having screenshots in the window, model-predicted risk was comparable regardless of completion status.

#### Follow-Up Interpretation

**Figure 4.**
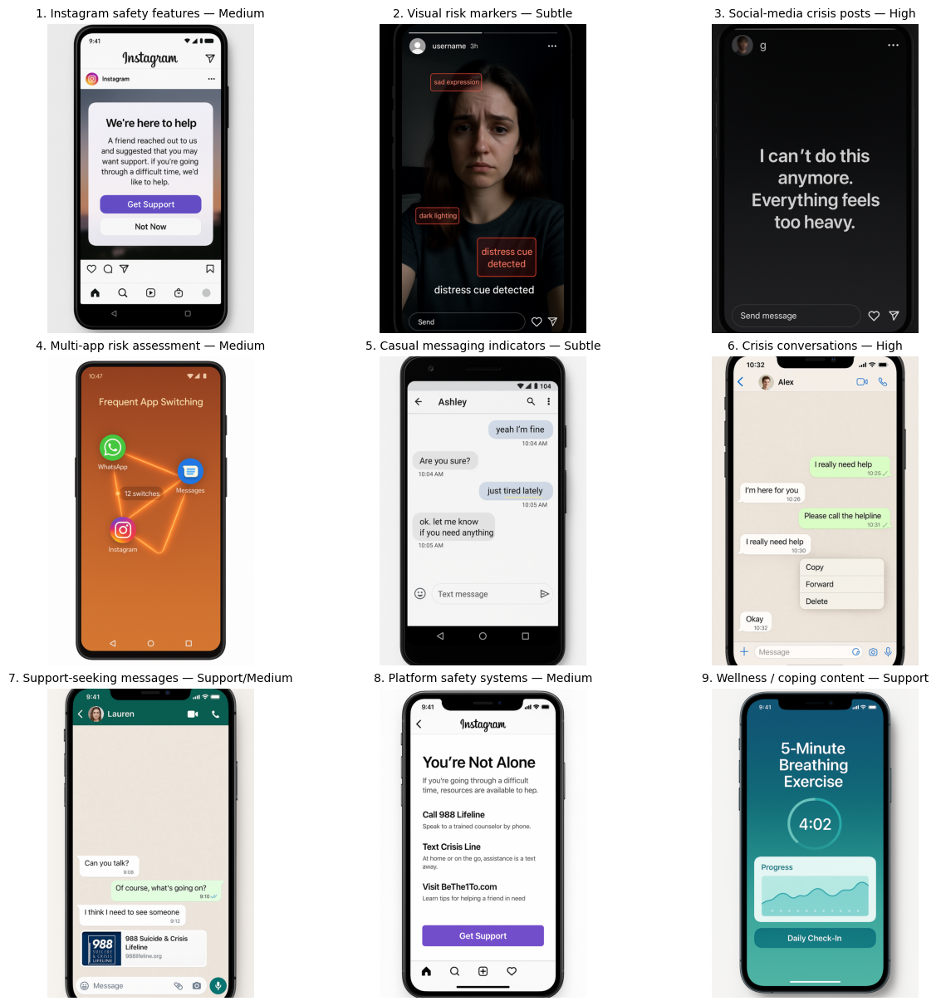
Synthetic smartphone mockups illustrating each topic. Images were generated using DALL-E 3 to visualize the user interface patterns identified by topic modeling and do not depict actual participant data. Topic labels represent general thematic clusters identified through automated analysis.

Applying this pipeline to the 1000 Qwen-VL–described screenshots produced a 9-topic solution in the LDA: (1) Instagram safety features, (2) platform safety systems (both graded Medium because they are crisis-adjacent but often triggered by platform logic), (3) crisis conversations, (4) social media crisis posts (graded high due to explicit crisis/help language), (5) multiapp risk assessment, (6) support-seeking messages (typically medium-support depending on the amount of explicit distress), (7) wellness/coping content (support), (8) visual risk markers, and (9) casual messaging indicators, which were retained as subtle topics capturing weaker but recurring contextual cues. In parallel, the NMF solution recovered highly similar structures but emphasized behavioral/interactional views of the same screens (eg, direct messaging, assessment interfaces, visual content with warnings, and emotional crisis chat), supporting the conclusion that 2 different topic-modeling families converged on the same underlying set of suicide-relevant screenshot patterns. The synthetic mockups (see Figure 4) mirrored these graded topics, a social post with a warning overlay for medium safety screens, back-and-forth messaging for high crisis chats, and a carded survey view for assessment topics, confirming that the latent topics mapped to concrete mobile UIs rather than to modeling artifacts.

## Discussion

### Principal Findings

The present study demonstrates that VLMs applied to smartphone screenshots predict short-term SI with clinically meaningful accuracy. This extends digital phenotyping beyond behavioral metadata [7,20,59]—GPS coordinates, accelerometer traces, and app usage logs—to the semantic content of digital experience itself; what people read, write, and view on their phones. Prior work applying natural language processing to suicide risk has largely relied on social media posts labeled as high-risk based on content or forum membership (eg, r/SuicideWatch; [60-62]), conflating the signal used for prediction with the outcome being predicted [63,64]. By contrast, the present approach predicts prospectively reported SI via EMA, providing a cleaner separation between predictor and criterion. Models trained on each person’s own history and evaluated with strict temporal holdouts reached EMA-level AUCs up to ~0.83, indicating that subtle, moment-to-moment variation in on-screen content carries an extractable signal about proximal risk.

A consistent finding was a performance gap between temporal and subject holdout prediction. Personalized models calibrated to an individual’s baseline were markedly more accurate than models applied to new individuals, aligning with the nonergodic character of many psychological processes [27,65] and consistent empirical findings in digital phenotyping [28,66]. This dissociation between model performances suggests a 2-stage clinical architecture. While complex VLMs failed to generalize across individuals (AUC≈0.50), simple lexical features yielded modest between-person discrimination (AUC=0.615). This implies that universal screening may rely on coarse, “nomothetic” signals (eg, specific crisis keywords), while precision monitoring requires high-capacity, “idiographic” models (ie, VLMs; [67]) that learn the subtle, pixel-level context of a specific patient’s digital life.

The stark contrast between temporal holdout (AUC≈0.83) and subject holdout (AUC≈0.50) prediction challenges the prevailing ’universal biomarker’ assumption in digital psychiatry [39]. Our results suggest that digital indicators of suicide risk are highly idiosyncratic; a specific app or interaction style that indicates risk for one patient may be benign for another. Consequently, clinical deployment should not rely on static, universal risk calculators, but rather on JITAIs that use a “warm-start” calibration period [29]. This allows the model to learn patient-specific baselines before triggering active interventions. Given the low base rate of suicidal thoughts and behaviors, effective systems will likely combine idiographic and nomothetic cues, particularly for rarer events such as suicidal planning or attempts, where purely person-specific signals may be sparse.

Comparing modeling approaches, aggregating predictions across the pre-EMA window outperformed single-screenshot decisions, underscoring that temporal patterns carry more signal than isolated frames. Image-based models significantly outperformed text-based models, suggesting that visual context, layout, app interface, and media content capture information beyond what OCR-extracted text alone provides. Among vision-language architectures, the smaller LFM2 matched Qwen at the screenshot level but showed attenuated EMA-level performance, indicating a potential tradeoff where smaller models capture frame-level signal but lose coherence when predictions are aggregated.

The near-chance performance of Mental-FLAN-T5 highlights the domain specificity of transfer learning in digital phenotyping. Mental-FLAN-T5 was instruction-finetuned on Reddit posts where users explicitly narrate psychological distress in coherent, first-person text. Screenshot OCR, by contrast, yields fragmented streams of UI elements, notifications, and app content that rarely contain explicit mental health language. This suggests that “text” is not a monolithic modality; models trained on one text source may not generalize to passive text streams without task-specific adaptation. The substantial improvement observed with fine-tuned models in the present study reinforces this conclusion and argues against relying on off-the-shelf mental health NLP tools for novel passive sensing applications. This highlights a fundamental “domain gap” in computational psychiatry. Models trained on performative social media posts (eg, r/SuicideWatch) fail to recognize the fragmented, multimodal reality of localized distress (eg, viewing platform safety pop-ups, navigating wellness apps, or ambiguous casual messaging).

To probe what drives model predictions, we applied topic modeling to VLM-generated screenshot descriptions. The resulting clusters aligned with clinically meaningful content, crisis resources, self-harm search behavior, late-night messaging, and platform safety interventions [16,18,68,69]. This suggests the models are not exploiting spurious artifacts but responding to content plausibly linked to acute distress. While not a formal explanation method, this analysis provides preliminary evidence that predictions are interpretable in terms recognizable to clinicians.

### Strengths and Innovation

To our knowledge, this is the first application of VLMs to passively collected smartphone screenshots for momentary suicide risk prediction. The dataset comprises ~7.5 million screenshots (~2.5 million within 2 hours of EMAs) from 70 high-risk participants, substantially larger than typical intensive longitudinal samples. By learning directly from pixel and text content rather than hand-engineered proxies, the approach captures information inaccessible to conventional sensor streams. Our evaluation used deployment-consistent temporal splits, and we found that window-level aggregation outperformed single-screenshot decisions, suggesting that patterns over time, not isolated screens, carry most signal.

### Key Limitations and Fairness

A principal limitation is the sample size at the person level (n=70). Although the dataset provided substantial power for within-person relations, we were severely underpowered to test whether relationships differ across demographic or socioeconomic subgroups. This is concerning given evidence that passive-sensing features can have population-specific associations with mental health; for example, [70] found mobility patterns that were protective for used/insured individuals paradoxically predicted higher depression risk for unemployed, uninsured, and low-income groups. Given that our sample was predominantly White (84.8%) and recruited from a single Midwestern region, the “digital phenotypes” discovered here (eg, specific wellness apps or communication styles) may reflect cultural norms that do not generalize. Future work must validate these semantic signals in diverse cohorts to ensure algorithmic equity. This limitation also constrains interpretation of subject holdout performance; with approximately 35 participants per split, we cannot distinguish whether near-chance between-person prediction reflects fundamental idiosyncrasy of digital behaviors or insufficient training data to learn cross-person regularities. Larger multisite samples are needed to adjudicate.

Additionally, we only analyzed screenshots linked to completed EMAs and did not explicitly model the missingness of the EMA outcome. Prior work shows higher phone use predicts higher EMA response [17] and that response rates vary with study characteristics (eg, decline with longer inclusion) [71], implying that nonresponse is predictable from vision/language features and study design. In related analyses (unpublished data), we found no evidence that capture degraded over time. This high adherence rate (>99% retention of capture capability) suggests that, despite the intrusive nature of continuous screenshotting, high-risk clinical populations may find “passive” surveillance more acceptable than the burden of active logging, provided (as in this study) strict privacy protocols are transparently communicated.

Future work should model EMA missingness jointly with risk (eg, selection or pattern-mixture approaches) or leverage screenshot-derived features for imputation/reweighting so that performance estimates reflect the full user experience. Our primary outcome was SI reported via EMA, not suicidal behavior; most ideation does not progress to action [72], making these models better suited as just-in-time intervention triggers (ie, targeting suicidal thinking; [73]) than standalone risk predictors. Finally, computational constraints inherent to fine-tuning foundation models on millions of images precluded extensive hyperparameter search, cross-validation, and sensitivity analyses (eg, varying the pre-EMA time window). Results should be interpreted as proof-of-concept under a specific configuration rather than optimized performance.

We examined whether EMA missingness could bias performance estimates. In a companion analysis (unpublished data), screenshot availability during EMA nonresponse showed no associations with suicide risk dynamics (mean levels, variability, and instability), suggesting non-selective coverage. Additionally, we trained models to predict EMA completion from temporal features and screenshot content; discrimination was modest (AUC=0.60 for temporal baseline, 0.66 with VLM), indicating that missingness is only weakly predictable from the features used in SI prediction. Together, these findings suggest that the ~31% missing EMAs are unlikely to substantially bias performance estimates, though future work should consider joint modeling approaches.

### Toward Privacy-Preserving Deployment

Although adequate predictive accuracy relied on within-individual fine-tuning, full on-device training of VLMs is not currently feasible on consumer smartphones due to memory, energy, and training time constraints. In this study, fine-tuning—even for compact models such as LFM2-VL—required GPU acceleration and large-scale batched optimization, exceeding the capabilities of mobile hardware. However, inference-only deployment of smaller VLMs is already feasible on modern devices, and our results show that reduced-capacity models retain strong within-person predictive performance. In practice, personalization could occur during an initial calibration phase using secure cloud-based resources, after which inference can be performed entirely on-device without transmitting raw screenshots. Alternative strategies, such as warm-start calibration, lightweight adapter tuning, or federated learning, may further reduce computational and privacy burdens while preserving individual-specific sensitivity.

Continuous screenshot capture is intrinsically sensitive, requiring multiple layers of technical safeguards. The smaller LFM2-VL model matched or exceeded larger architectures on temporal holdout prediction, enabling a privacy-first architecture where sensitive pixel data never leaves the patient’s phone; only computed risk scores would be transmitted to clinicians. Additional safeguards include ephemeral processing, where screenshots are held only in volatile memory during inference and immediately discarded, preventing recovery even if the device is compromised. Content-aware redaction can automatically mask identifiable information (faces, names, and financial data) before any storage, and our topic modeling results suggest that clinically relevant signals concentrate in specific content categories (crisis conversations and platform safety warnings), potentially enabling selective capture that preserves predictive utility while minimizing data exposure. For applications requiring formal guarantees, differential privacy mechanisms could be integrated during model training and inference to prevent reconstruction of individual screenshots from model outputs.

Effective deployment also requires stakeholder co-design extending beyond technical safeguards [74,75]. Participants should have granular, reversible control over data sharing, specifying which content categories to monitor, at what temporal resolution, and for what duration, with transparent interfaces showing what data are captured and transmitted. Given documented disparities in passive sensing model performance across demographic groups [70], co-design processes must include diverse patient populations to ensure privacy-preserving systems do not inadvertently exacerbate health inequities. Balancing detection accuracy against false alarm burden, encryption for data in transit, and threshold co-design with end users and clinicians will be essential for responsible clinical translation.

### Clinical Deployment Considerations

Translating screenshot-based risk detection into clinical practice requires addressing the calibration limitations observed in this study. The substantial probability compression (calibration slope=4.71) means that model outputs do not correspond to true event probabilities; until calibration is improved through alternative aggregation strategies or sufficient individual-level calibration data, deployment systems should treat outputs as ordinal risk rankings rather than calibrated probabilities. We recommend that model outputs inform, not replace, clinical judgment: risk-event feeds could route to care-management dashboards where clinicians review alerts and determine appropriate outreach [20], with integration into existing clinical workflows remaining an important implementation challenge. The modular architecture demonstrated here, with separable feature extraction, temporal aggregation, and risk scoring, is compatible with JITAI frameworks [76], though integration with intervention delivery systems remains to be tested. From a model safety perspective, the fine-tuned models in this study output scalar risk probabilities rather than natural language, limiting the potential for amplifying self-harm content. Deployment in clinical systems should nonetheless incorporate content filtering on any model-generated text, restrict outputs to ordinal risk rankings rather than explanatory narratives, and require clinician-in-the-loop review before any intervention is triggered.

### Conclusions

Screenshot content predicts short-term SI with modest but reliable accuracy. Fine-tuned VLMs outperformed zero-shot approaches, and temporal holdout evaluation yielded stronger discrimination than subject holdout generalization. These results establish that passively captured screen content, despite its fragmentary, noisy nature, carries an extractable signal about proximal suicide risk.

## Supplementary material

10.2196/90581 Multimedia Appendix 1 Model prompts used for zero-shot suicide risk scoring (Llama 3.2 11B Vision-Instruct and Qwen3-30B-A3B), vision-language model interpretation of high-risk screenshots (Qwen-VL), and synthetic smartphone mockup generation (DALL-E 3).

## Abbreviations

AUC: area under the receiver operating characteristic curve
AUPRC: area under the precision-recall curve
EMA: ecological momentary assessment
HIPAA: Health Insurance Portability and Accountability Act
JITAI: just-in-time adaptive intervention
LDA: Latent Dirichlet Allocation
LFM2: Liquid AI Foundation Model
LoRA: Low-Rank Adaptation
NMF: nonnegative matrix factorization
OCR: optical character recognition
SI: suicidal ideation
VLM: vision-language model
