# Supplementary material for: Predicting Momentary Suicidal Ideation From Smartphone Screenshots Using Vision-Language Models: Prospective Machine Learning Study
Source: JMIR Ment Health. 2026 Apr 8;13:e90581. doi: 10.2196/90581 (PMC13061109; doi:10.2196/90581)
Supplement: Multimedia Appendix 1 [file mental-v13-e90581-s001.docx]

# Supplemental Material

## Zero-Shot Learning

We employed Llama 3.2 11B Vision-Instruct, a multimodal large language model, to directly assess suicide risk from smartphone screenshots. The model was prompted to analyze each screenshot as a suicide researcher, evaluating multiple risk indicators including suicide-related language, signs of social isolation, negative affect, substance use, and poor sleep patterns. Specifically, we used the following prompt:

"As a suicide researcher, analyze the provided smartphone screenshot for indicators of suicide risk. Assess for suicide-related language, signs of social isolation, or other risk factors, that may include, but is not limited to, indicators of strong negative affect, substance use, poor sleep, or other negative states. In your rating, give more weight to text generated by the owner of the smartphone, as opposed to passively viewed text or images. On a scale from 0 to 10, where 0 indicates no risk and 10 indicates extreme risk, rate this image for suicide risk. The score I would give is".

This zero-shot approach leverages the model's pretrained knowledge to generate risk scores without requiring task-specific fine-tuning, processing screenshots in batches of 32 with deterministic generation parameters (temperature=0.1, top_p=1.0, do_sample=False) to ensure consistent scoring across the screenshots.

For the Qwen-30B-A3B application, we used a different prompt:

"""System message for EMA-behavioral correlation learning"""

return """You are a multimodal assistant trained to identify subtle digital behavioral patterns that correlate with psychological states measured through ecological momentary assessments (EMA).

Your task is to learn behavioral signatures from smartphone usage patterns that predict momentary psychological states within 2-hour windows. Focus on:

- Usage timing and frequency patterns

- App transition behaviors and digital habits

- Social interaction patterns and engagement levels

- Content consumption behaviors and preferences

- Digital attention and focus patterns

- Multitasking and app-switching behaviors

The correlation between digital behavior and psychological states is often subtle and pattern-based rather than content-explicit."""

"""System message for EMA-behavioral correlation learning"""

return """You are a multimodal assistant trained to identify subtle digital behavioral patterns that correlate with psychological states measured through ecological momentary assessments (EMA).

Your task is to learn behavioral signatures from smartphone usage patterns that predict momentary psychological states within 2-hour windows. Focus on:

- Usage timing and frequency patterns

- App transition behaviors and digital habits

- Social interaction patterns and engagement levels

- Content consumption behaviors and preferences

- Digital attention and focus patterns

- Multitasking and app-switching behaviors

The correlation between digital behavior and psychological states is often subtle and pattern-based rather than content-explicit."""

## Follow-Up Interpretation Prompt.

We used the following prompt for the Qwen-VL model applied to the top 1000 predictions.

"""You are an expert psychologist analyzing smartphone screenshots for suicide risk indicators. Please provide a detailed description of this image, focusing on:

1. **App/Platform**: What app or platform is shown? (e.g., Instagram, TikTok, Messages, WhatsApp, etc.)

2. **Content Type**: What type of content is displayed?

- Social media posts/stories

- Text messages/chats

- Web pages/articles

- Photos/videos

- Other app interface

3. **Suicide-Related Language**: Are there any explicit or implicit references to:

- Suicide ideation, self-harm, or death

- Depression, hopelessness, or despair

- Crisis keywords or phrases

- Mental health struggles

4. **Visual Cues**: Describe any concerning visual elements:

- Dark or disturbing imagery

- Self-harm related content

- Crisis hotline numbers or mental health resources

- Warning messages or intervention screens

5. **Social Context**: If applicable, describe:

- Conversations about mental health

- Support seeking behavior

- Crisis communication patterns

- Interactions with others about emotional distress

6. **Interface Elements**: Note any specific UI elements:

- Search terms related to suicide/self-harm

- Mental health apps or resources

- Crisis intervention features

- Warning dialogs or safety features

Please be thorough and specific in your analysis. Even if no obvious suicide-related content is visible, describe what you see as it may contain subtle indicators the AI model detected."""

## DALL-E 3 Prompt

For clarity purposes, we do not provide the full prompt, which is available upon request. To ensure ethical integrity, we used no real participant data, labeled all images as “SYNTHETIC – RESEARCH ONLY,” and followed IRB and privacy safeguards (e.g., placeholder text, generic names, no identifiable elements). Each image was rendered in a 9:16 mobile aspect ratio at 1080×1920 px resolution with a photorealistic smartphone-UI style and explicit negative prompts to avoid visual artifacts. For each topic—spanning Instagram safety features, visual risk markers, crisis posts, multi-app risk assessment, casual messaging indicators, crisis conversations, support-seeking messages, platform safety systems, *and* wellness/coping content—we specified optimized text prompts describing platform-authentic UI elements, color palettes, and interaction contexts. We generated 2–3 variations per topic and selected the clearest, most realistic renderings for inclusion. All final images were manually screened to confirm accurate UI proportions, legible text, and appropriate ethical presentation.
